# Selected immunoendocrine and performance adaptations to upper-body plyometric training and β-alanine supplementation in male swimmers

**DOI:** 10.1080/15502783.2026.2679050

**Published:** 2026-05-31

**Authors:** Hui Gao, Xuemei Yu, Zhiyong Guo

**Affiliations:** a School of Physical Education, Xi’an University of Finance and Economics, Xi'an, People’s Republic of China

**Keywords:** β*-*alanine, buffering capacity, immune function, plyometric training, swimming performance

## Abstract

**Background:**

Upper-body plyometric training (PT) enhances neuromuscular performance and power production. However, its application in swimming and its potential interaction with β-alanine (BA) supplementation remain largely unexplored. Therefore, this study investigated the combined effects of upper-body PT and BA supplementation on performance and immunoendocrine adaptations in trained male swimmers.

**Methods:**

Thirty trained male swimmers (age: 25.7 ± 2.6 years; height: 1.83 ± 0.07 m; body mass: 81.6 ± 3.2 kg) were randomly assigned to one of three groups: PT + BA, PT + placebo (PL), or control (CON). *Participants in the PT groups completed an 8-week upper-body PT program (2 sessions·week⁻¹).* Each session consisted of upper-body plyometric exercises performed for 3–4 sets of 12–16 repetitions with 120 s rest between sets. *Participants in the supplementation groups ingested* β*-alanine (4.8 g·day⁻¹ divided into six 0.8 g doses) or a matched placebo daily throughout the intervention.* Assessments conducted before and after the intervention included medicine ball throw (MBT), push-up endurance, one-repetition maximum (1RM) bench press, swim ergometer peak and mean power output (PPO and MPO), and 50-, 100-, and 200-m freestyle performance. Blood samples were also collected pre- and post-intervention to determine testosterone, cortisol, and immunoglobulin A (IgA) concentrations.

**Results:**

Both PT groups showed significant improvements in all performance outcomes compared with baseline (all *p* < 0.05), with effect sizes ranging from small to moderate. *Post hoc analyses revealed a significant group × time interaction for MBT (p = 0.045), push-up endurance (p = 0.041), PPO (p = 0.026), and MPO (p = 0.033), indicating greater improvements in the PT + BA group than in the PT + PL group. In addition, following the training intervention, the PT + BA group showed significant changes in testosterone (p = 0.011), cortisol (p = 0.028), and IgA (p = 0.042) concentrations compared with the PT + PL group.*

**Conclusions:**

Eight weeks of upper-body PT significantly enhanced upper-body power, anaerobic capacity, and swimming performance in trained male swimmers. BA supplementation further amplified these training-induced adaptations and promoted a more favorable immunoendocrine adaptation profile. *These findings suggest that combining upper-body PT with* β*-alanine supplementation may provide synergistic benefits for performance development and physiological recovery in competitive swimmers.*

## 1. Introduction

Swimming performance is underpinned by the ability to generate high rates of force development during key race segments, including starts, turns, and maximal-intensity swimming [1]. In competitive swimming, particularly across middle-distance events (e.g. 100–400 m), athletes are exposed to substantial metabolic demands, with a pronounced contribution from the anaerobic glycolytic system to support rapid power production and the maintenance of repeated high-intensity efforts [2]. Moreover, muscular power represents a key determinant of swimming performance, as elevated levels of upper-body power have been consistently associated with superior stroke mechanics and propulsion efficiency [3]. Consequently, the implementation of an appropriately designed training program is essential for optimizing both physiological and physical performance in swimmers. Such programs should prioritize the development of explosive muscular power and glycolytic capacity to maximize competitive performance [4].

Upper-body plyometric training (PT) represents a well-established and effective approach for enhancing neuromuscular performance across a variety of athletic populations [5,6]. Substantial evidence supports its efficacy in improving power output, rate of force development, and overall sport-specific performance in athletes [7,8]. However, in contrast to the extensive literature on lower-body plyometric interventions, the application of upper-body PT in swimming has received little scientific attention despite the dominant role of the upper extremities in swimming propulsion and stroke generation. Given that front-crawl swimming relies heavily on upper-body force production and rapid stretch-shortening cycle actions during the pull-through phase, upper-body PT may provide a sport-specific stimulus capable of enhancing swimming performance. Nevertheless, the efficacy of this training modality in swimmers remains largely unexplored. A recent systematic review by García-Carrillo et al. [5] identified a notable absence of empirical studies examining the influence of upper-body PT on performance adaptations in swimmers, underscoring the paucity of sport-specific evidence in this domain. Therefore, an investigation into the effects of upper-body PT on the physiological and performance profiles of male swimmers would substantially extend current knowledge and offer practical, evidence-based recommendations for strength and conditioning professionals in this sport.

The implementation of PT imposes substantial metabolic stress, characterized by elevations in blood lactate and hydrogen ion concentrations, which may accelerate the onset of fatigue and impair muscle contractile function and energy metabolism [7,8]. Such disturbances have the potential to attenuate the sustainability of performance adaptations following the training period [9]. Consequently, the maintenance of intramuscular pH homeostasis during PT is critical for sustaining high-intensity exercise capacity and optimizing training volume and adaptation [9]. *Accordingly, nutritional strategies capable of enhancing buffering capacity may potentiate the adaptive responses induced by PT [10].* In light of these metabolic demands, β-alanine (BA) supplementation may represent an effective ergogenic strategy, as BA augments intramuscular carnosine content, thereby enhancing muscle endurance capacity [10]. *Through increasing intramuscular carnosine concentrations, BA supplementation enhances intracellular buffering capacity and attenuates exercise-induced acidosis, which may delay fatigue onset during repeated high-intensity muscular contractions [10–12].* Notably, BA-induced increases in carnosine occur in both fast- and slow-twitch muscle fibers, improving intracellular buffering capacity, delaying fatigue onset, and potentially maximizing training-induced performance adaptations [11,12]. Therefore, combining BA supplementation with upper-body PT may produce synergistic effects by enabling swimmers to tolerate greater training demands, maintain higher movement quality during explosive exercise, and enhance neuromuscular and metabolic adaptations over time [9–13].

Beyond performance-related outcomes, the monitoring of immunoendocrine responses is critical for optimizing training load and minimizing maladaptive consequences in swimmers. Elevated training stress triggers complex physiological and biochemical responses, underscoring the importance of assessing key markers such as immunoglobulin A, testosterone, and cortisol to guide training prescription and reduce the risk of overreaching or overtraining [13]. Nevertheless, the effects of this specific upper-body PT approach on immunoendocrine regulation and adaptive optimization in swimmers remain unexplored.

Although BA supplementation is well established for its ergogenic effects on athletic performance and evidence supports the positive transfer of PT to performance outcomes [9–12], the combined influence of upper-body PT and BA supplementation on physical and physiological performance as well as immunoendocrine adaptations in swimmers remains unknown. In particular, no studies have examined these interactions in male swimmers over a training period. Therefore, the aim of the present study was to investigate the effects of an 8-week upper-body PT program combined with BA supplementation on anaerobic power, maximal strength, muscular power, push-up endurance, 50-m, 100-m, and 200-m freestyle performance, as well as immunoendocrine adaptations in trained male swimmers. It was hypothesized that the combined PT + BA intervention would induce greater improvements in physical performance and more favorable immunoendocrine adaptations compared with PT alone or the control condition.

## 2. Materials and methods

### 2.1. Participants

Thirty young male swimmers specializing in short‑ and middle‑distance freestyle events volunteered to participate in the study and were randomly assigned to two PT groups and a control (CON) group (Table 1). The participants were aged 25.7 ± 2.6 years, with a body height of 1.83 ± 0.07 m, and body mass of 81.6 ± 3.2 kg. The swimmers had 7 ± 2 years of competitive swimming experience (training age) and trained at least four sessions per week at a regional/national competitive level, classifying them as trained (i.e. Tier 2) according to the criteria proposed by McKay et al. [14]. To be eligible for inclusion, athletes were required to meet the following criteria: (1) prior experience with various forms of PT, with no engagement in such training during the three months preceding the study; (2) absence of any medical or orthopedic conditions that could compromise participation or performance, as confirmed by evaluation from a sports medicine physician; and (3) no consumption of any supplements, specifically BA, within the six months preceding the study. All participants received comprehensive information regarding the potential risks and benefits associated with the study prior to participation. The experimental procedures were approved by the University's Ethics Committee and were conducted in accordance with the latest revision of the Declaration of Helsinki.

**Table 1.** Participants characteristics'.

		PT + BA (*n* = 10)			PT + PL (*n* = 10)			CON (*n* = 10)		
Characteristic		mean	±	SD	mean	±	SD	mean	±	SD
Age	(y)	25.3	±	2.8	25.7	±	2.5	26.2	±	2.7
Height	(m)	1.82	±	0.07	1.85	±	0.08	1.84	±	0.08
Body mass	(kg)	81.0	±	3.6	81.9	±	3.5	82.0	±	2.6

### 2.2. Sample size estimation and randomization

An a priori sample size calculation was performed using G*Power software (version 3.1.9, Universität, Düsseldorf, Germany). Based on previous studies examining the effects of PT on performance outcomes in swimmers [6], an effect size of f = 0.21 was assumed. With an alpha level set at 0.05 and statistical power of 0.80, the minimum required sample size was determined to be 9 participants. To accommodate potential participant dropout during the data collection phase, the final sample size was increased to 10 participants per group. Participants were randomly allocated to one of the three groups (PT + BA, PT + PL, or CON) using a computer-generated simple randomization procedure with a 1:1:1 allocation ratio. The randomization sequence was prepared by a researcher not involved in the testing or training supervision procedures to minimize allocation bias. Group assignments were concealed in sealed opaque envelopes, ensuring allocation concealment and reducing the risk of selection bias. The allocation process was therefore unpredictable to both the investigators and the participants.

### 2.3. Study design

To determine the effectiveness of BA supplementations on the efficacy of PT adaptive responses in physical and physiological variables as well as immunoendocrine responses, a 10-week randomized controlled longitudinal study was conducted. The study timeline comprised a pre-intervention testing week, followed by 8 weeks of BA and PT intervention, and a post-intervention testing week. Prior to baseline assessments, all participants attended a laboratory familiarization session, during which they were introduced to the study objectives, testing protocols, and training procedures. Anthropometric characteristics were also assessed during this session. Performance and physiological testing were conducted across two separate sessions. On the first day, athletes completed assessments of muscular power (medicine ball throw [MBT]) and endurance (push-up) and strength (one repetition maximum [1RM] of bench press). The second session included evaluations of anaerobic power using the swim ergometer test as well as 50-m, 100 m and 200-m freestyle performance tests. A minimum 20-min recovery period was provided between tests within each session, and a 72-h interval separated the two testing days to minimize residual fatigue. Throughout the 8-week intervention, participants consumed either the assigned PL or BA supplements in a double-blind manner alongside their regular swimming training. In contrast, the CON did not receive any supplementation and completed only the prescribed swimming training program. All testing procedures were performed in the afternoon (16:00–18:00 h) to control for diurnal variation. To examine training-induced immunoendocrine adaptations, venous blood samples were collected in the morning hours under fasting conditions before the onset of the training intervention and after completion of the 8-week intervention period.

### 2.4. Supplementation

Participants assigned to the BA supplementation group consumed 4.8 g·day⁻¹ of β-alanine (Pure Organic Ingredients, USA). The daily dose was administered as six equal 0.8 g servings ingested at 2-h intervals, consistent with the protocol described by Trexler et al. [15]. The PL group received an equivalent daily dose of maltodextrin. Both supplements were encapsulated in identical opaque capsules and ingested with juice to maintain double-blind conditions. Neither the participants nor the investigators were aware of the capsule contents until completion of the study. Compliance with the supplementation protocol was monitored through weekly adherence logs, verbal confirmation during training sessions, and the return of empty supplement containers at the end of each week. Supplement packets were distributed and counted weekly to verify adherence throughout the intervention period. In addition, participants were asked at the conclusion of the study to indicate whether they believed they had received beta-alanine or placebo in order to evaluate the effectiveness of the blinding procedure. A sustained-release formulation of β-alanine was employed to attenuate paresthesia commonly associated with BA ingestion. The inclusion of a PL group receiving maltodextrin was intended to control for expectancy and psychological effects associated with supplementation, thereby enabling isolation of the physiological effects of BA. This placebo-controlled, double-blind design is widely recognized as the methodological gold standard for evaluating the efficacy of beta-alanine supplementation and has been recommended in previous supplementation research [13].

### 2.5. Training intervention

All athletes completed a four-day-per-week swimming training program during the preparatory phase. Each session lasted approximately 110–120 minutes and consisted of a 15-minute warm-up, 85–95 minutes of main training activities, and a 10-minute cool-down. The training program consisted of moderate-intensity front-crawl sets performed with short recovery intervals, along with technical training in the individual medley. Each session involved a total training volume of approximately 4800 ± 300 m. Participants in the PT groups completed two training sessions per week over an 8-week intervention period. In contrast, the CON group did not participate in any structured training intervention and continued their habitual training routines. Each PT session lasted approximately 60–70 minutes and was conducted in the afternoon (16:00–18:00 h). Sessions began with a standardized 15-minute warm-up. The PT program was structured according to the principle of progressive overload and consisted of multiple exercises and sets, following established guidelines reported in previous research (Table 2). The entire training program was supervised by an experienced strength and conditioning coach to ensure adherence to prescribed training methods and maximal effort during all trials [4,5].

**Table 2.** PT program.

Exercises	Weeks	Sets	Reps
Medicine ball chest pass*	1	3	12
Medicine ball overhead throw	2	3	13
Plyo push-up with knee ground touch	3	3	14
Drop medicine ball in lying position	4	3	15
	5	4	13
	6	4	14
	7	4	15
	8	4	16
Rest between trials (sec)	3	3	3
Rest between sets (sec)	120	120	120

^*^ week 1 and 2, 3kg, week 3 and 4, 4kg, week 5 and 6, 5kg, week 7 and 8, 6kg.

### 2.6. Testing procedure

All assessments were conducted across standardized testing sessions under controlled environmental conditions (i.e. controlled laboratory, 27–29 °C; 40–45% relative humidity). Prior to each testing session, participants were instructed to adhere to standardized pre-assessment guidelines, including obtaining at least 8 h of sleep, consuming a carbohydrate-rich diet (~65% of daily caloric intake), and abstaining from alcohol, caffeine, and other ergogenic aids; compliance was verified via personal interviews. All measurements were performed at similar times of day to minimize circadian variability, and identical procedures were applied at both pre- and post-intervention assessments.

#### 2.6.1. Anthropometric measurements

Participants' standing height was measured using a wall-mounted stadiometer (SECA, Germany; ±0.1 cm), and body mass was assessed with a calibrated electronic digital scale (BEURER, Germany; ±0.1 kg). Body fat percentage was determined using a bioelectrical impedance analyzer (InBody 270; Biospace Co., Ltd., Korea).

#### 2.6.2. Muscular power and endurance measurements

Upper-body muscular power was assessed using a seated MBT. Participants sat on a chair holding a 3-kg medicine ball with both hands, arms flexed at 90° in front of the torso. They were instructed to throw the ball as far as possible without a backward or forward countermovement. The best of three trials, separated by 30-s rest intervals, was recorded for analysis [16]. The push-up test to maximal repetition was used to evaluate upper-body muscular endurance. Participants assumed a standard push-up position, with hands placed under the shoulders, arms extended, legs together, and toes tucked. Each repetition required lowering the body until the elbows reached 90° of flexion and then returning to full arm extension while maintaining a straight line from the back through the legs. The test was ended when the participant paused, failed to maintain proper form, or was unable to achieve the required elbow angle on two consecutive attempts. The total number of correctly performed push‑ups was recorded [17].

#### 2.6.3. Muscular strength measurement

Upper‑body maximal strength was assessed using the bench 1RM test, performed in accordance with the guidelines of Kraemer and Fry [18]. Following a standardized warm-up, participants completed 3–5 sets of progressively increasing loads, with 2-min rest intervals between attempts, until their maximal lifting capacity was reached. All trials were performed under the supervision and control of two trained spotters to ensure participant safety and to prevent any external assistance during the lift. For each attempt, the barbell was lowered in a controlled manner until it lightly contacted the chest and then pressed upward to full elbow extension without bouncing or assistance. Any lift that failed to meet the prescribed technical criteria (e.g. incomplete range of motion, excessive trunk movement, or assistance from the spotters) was deemed invalid and excluded from analysis. The heaviest load successfully lifted with proper technique was recorded as the participant's 1RM.

#### 2.6.4. Anaerobic power measurement

Two maximal exercise assessments were performed on a stationary swim ergometer that had been calibrated according to the manufacturer's recommendations [19]. Before testing, participants completed a standardized warm-up protocol consisting of ten pulling repetitions with gradually increasing intensity, progressing from light effort to a submaximal level, as described in previous research [19]. The purpose of these tests was to quantify peak power output (PPO) and average power output (APO). PPO was defined as the highest power value attained during a 6-s maximal sprint, while APO represented the mean power output sustained over a 30-s all-out effort. Both sprint protocols were conducted with no external resistance to ensure unrestricted force production and to allow participants to express maximal power throughout the duration of each test [20].

#### 2.6.5. Freestyle swimming performance

Freestyle swimming performance tests were conducted in a 25-m indoor swimming pool, with water temperature maintained at approximately 27.5 °C over three short-course freestyle distances (50-m, 100-m, and 200-m). Before each trial, participants completed a standardized warm-up as described by Neiva et al. [21]. Trials began with a dive start on an audio signal. Two experienced timekeepers recorded the time for each distance using handheld stopwatches (SEIKO S120-4030, Tokyo, Japan); the average of the two measurements was taken as the trial time. Stroke rate was determined over the middle 10 m of the pool by timing three complete stroke cycles with a stopwatch and converting the result to cycles per minute. All tests were conducted under identical environmental and pool conditions before and after the intervention period.

#### 2.6.6. Immunoendocrine measurements

Blood samples (10 mL) were drawn from the antecubital vein in the morning after an overnight fast of at least 8 hours. Sampling occurred 48 hours before the first training session and 48 hours after the final session of the 8-week intervention. The samples were centrifuged at 3,000 rpm for 15 minutes at 4 °C, and the resulting serum was aliquoted and stored at −80 °C until analysis. Serum concentrations of testosterone and cortisol were quantified using commercially available enzyme-linked immunosorbent assay (ELISA) kits (Monobind, Inc., Lake Forest, CA, USA), with an intra-assay coefficient of variation (CV) of <10%. Serum immunoglobulin A (IgA) levels were measured on a Hitachi analyzer using a MININEPH™ Human IgA Kit (Birmingham, UK). Selective IgA deficiency was defined as a serum IgA concentration below 50 mg/L.

### 2.7. Diet control

To minimize the potential influence of nutritional status on performance outcomes, the dietary control measures were implemented. Participants were strictly prohibited from consuming any nutritional supplements or performance-enhancing substances throughout the study period. In addition, all participants were instructed to maintain a detailed three-day dietary record. Dietary data were subsequently analyzed using Nutritionist Pro™ software (version IV) to determine total energy intake as well as macro- and micronutrient composition. Analysis revealed comparable caloric intake across participants, with mean dietary composition consisting of approximately 20% protein, 65% carbohydrates, and 15% fat.

### 2.8. Statistical analysis

Data are reported as mean ± standard deviation (SD). The assumption of normality was verified for both pre- and post-intervention values using the Shapiro–Wilk test, and homogeneity of variances was assessed with Levene's test. Group differences over time were examined using a two-way repeated-measures analysis of variance (ANOVA) with factors of time (2 levels: pre and post) and group (3 levels: PT + BA, PL + PL and CON). When significant effects were detected, Bonferroni-adjusted post-hoc comparisons were performed. Effect sizes (ES) with corresponding 95% confidence intervals (CIs) were calculated to quantify the magnitude of training-induced changes. ESs were interpreted according to the criteria proposed by Hopkins et al. [22], whereby values <0.2 were considered trivial, 0.2–0.6 small, 0.6–1.2 moderate, 1.2–2.0 large, 2.0–4.0 very large, and >4.0 nearly perfect. Individual percentage changes for each outcome were calculated as ∆% = [(post − pre)/pre] × 100. Additionally, between group differences in percentage changes (∆%) were analyzed using a one-way ANOVA and also Bonferroni post-hoc test. Statistical significance was set at *p* < 0.05. All analyses were conducted using SPSS statistical software (version 21.0; SPSS Inc., Chicago, IL, USA).

## 3. Results

All participants completed the prescribed training and testing protocols, yielding a compliance rate of 100% as verified by individual attendance logs. No training- or testing-related injuries were reported during the study period. Furthermore, neither BA nor PL supplementation was associated with any gastrointestinal adverse events that confirmed by personal interview.

### 3.1. Within group changes

Following the 8-week intervention, both the BA and PL groups exhibited significant improvements from pre to post-testing (all *p* < 0.05), with ESs ranging from small to moderate across MBT, push-ups, 1RM strength, PPO, MPO, and freestyle performance (Tables 3–6). With respect to immunoenducrine responses, testosterone concentrations increased following the intervention in the BA group, whereas reductions were observed in the PL group. Conversely, cortisol levels decreased in the BA group but increased in the PL group over the training period. IgA remained unchanged in the BA group, while a significant post-intervention reduction was detected in the PL group (Table 7). In contrast, the CON group demonstrated no significant pre to post changes, with trivial ESs observed for all assessed variables.

### 3.2. Group × time interaction effects

**Figure 1.**
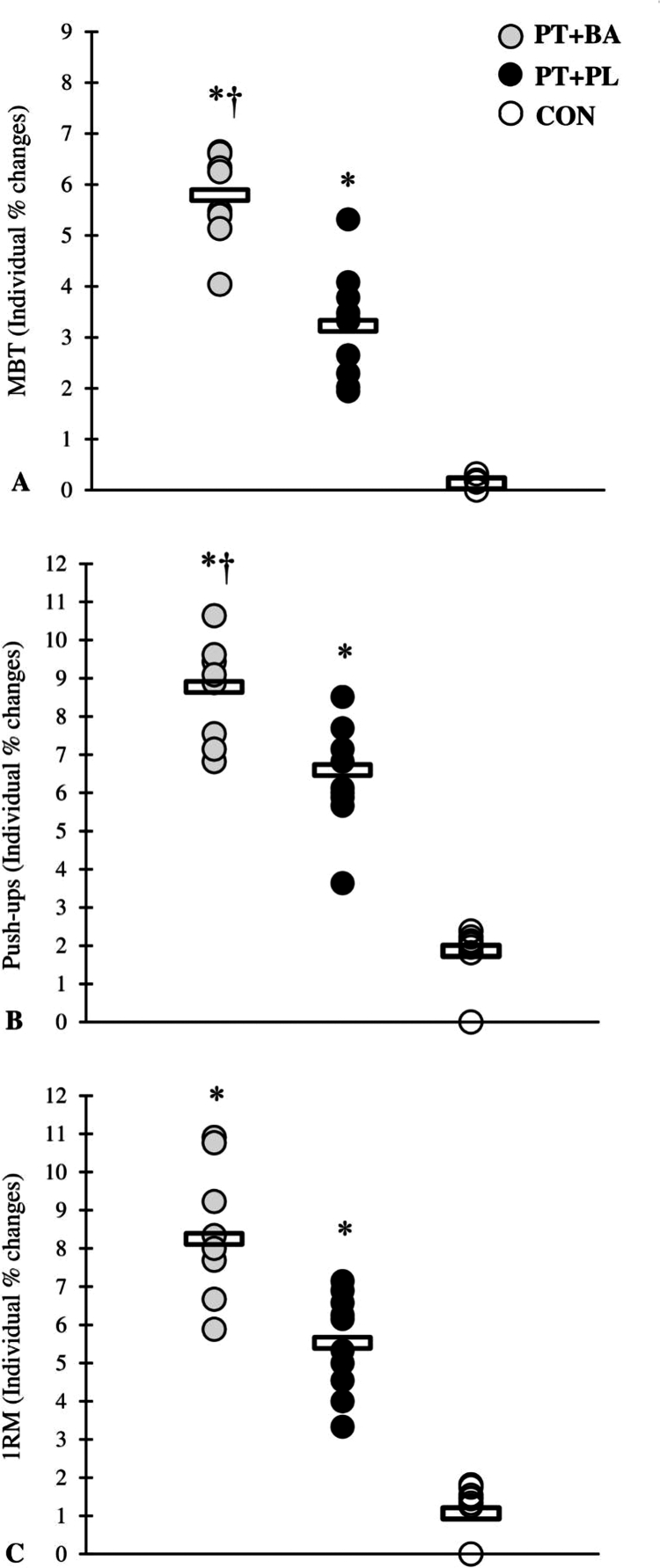
% changes in the physical performance through the 8-week intervention period. *denotes significant differences compared with the CON, †denotes significant differences compared with the PL.

**Figure 2.**
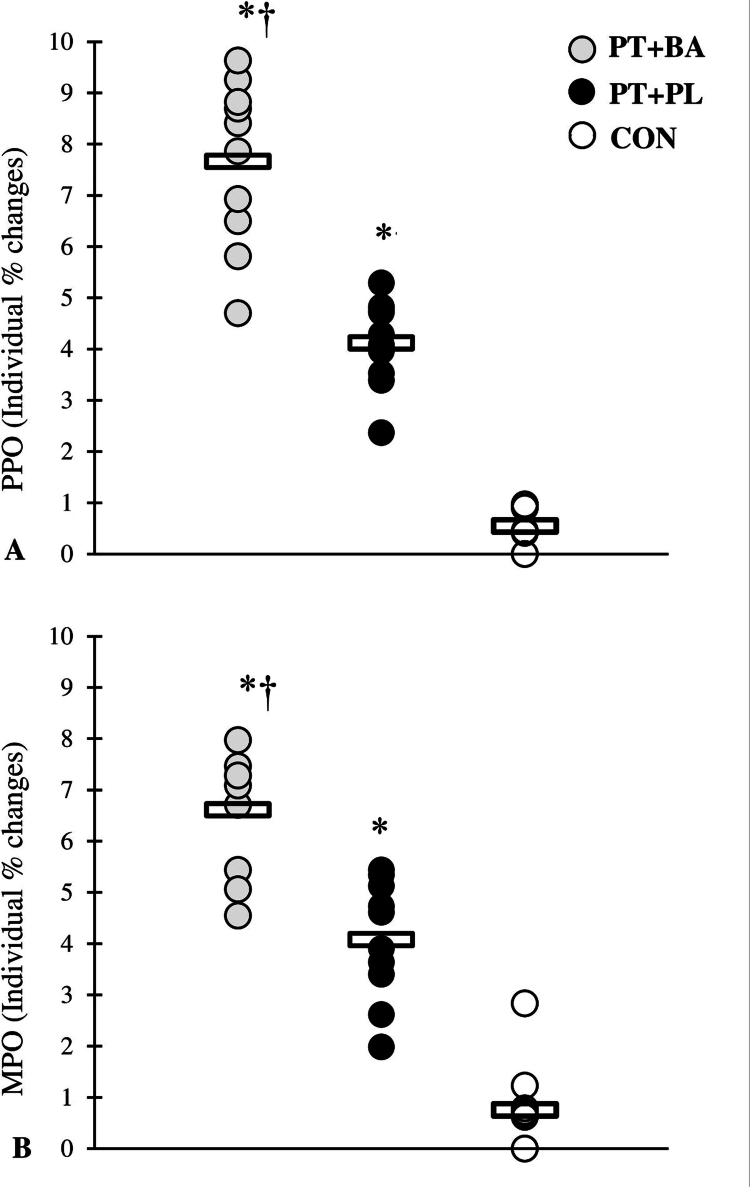
% changes in the power performance through the 8-week intervention period. *denotes significant differences compared with the CON, †denotes significant differences compared with the PL.

**Figure 3.**
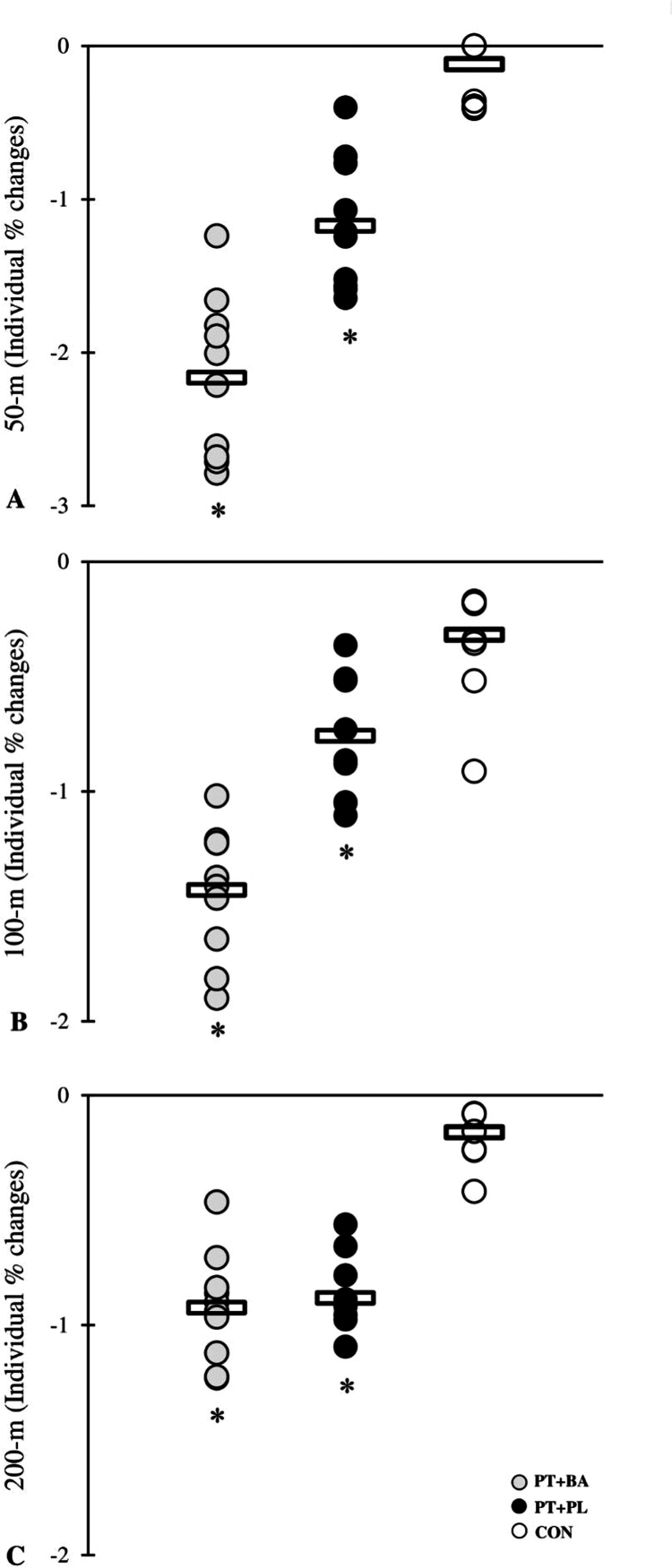
% changes in the swimming time performance through the 8-week intervention period. *denotes significant differences compared with the CON.

**Figure 4.**
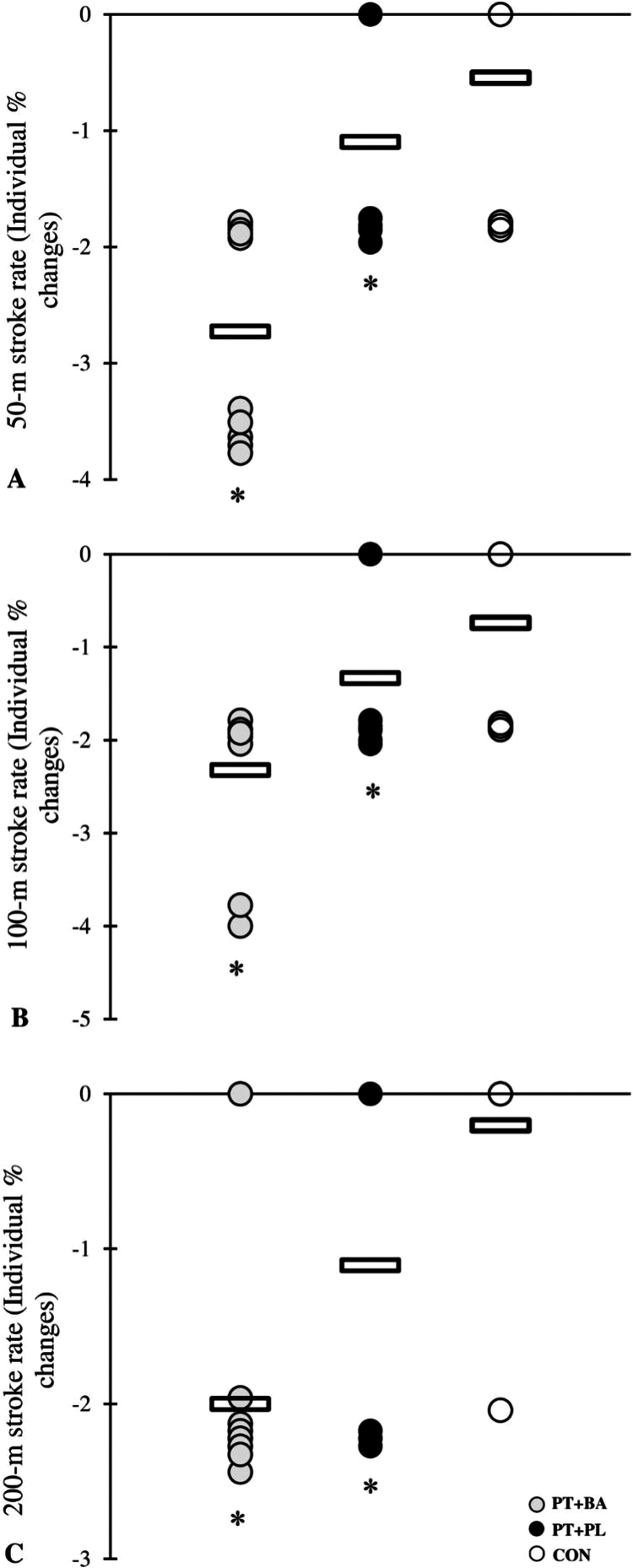
% changes in the swimming stroke rate performance through the 8-week intervention period. *denotes significant differences compared with the CON.

A significant group × time interaction was observed for all performance variables (all *p* ≤ 0.001), indicating differential adaptations among the training groups vs. CON group (Tables 3–6, Figures 1–4). The magnitude of the increase in testosterone was significantly greater in the BA group compared with the CON group following the intervention. Similarly, the reduction in cortisol was more pronounced in the BA group than in the CON group over the training period (Table 7).

**Table 3.** Changes in the physical performance variables from pre to post-training (mean ± SD).

Variable	Pre	Post	Significance	ES (95% CI)
MBT (cm)	*Time effect*			
PT + BA	591.8 ± 49.8	626.2 ± 54.4	*0.001*	0.63 (–0.27 to 1.53) Moderate
PT + PL	590.7 ± 51.0	610.1 ± 55.9	*0.001*	0.35 (–0.54 to 1.23) Small
CON	590.2 ± 49.5	591.0 ± 50.0	*0.864*	0.02 (–0.86 to 0.89) Trivial
*Post-test comparisons*	*Group x time effect, p = 0.001, n2p = 0.87*			
	PT + BA vs. CON PT + PL vs. CON PT + BA vs. PT + PL		*0.004*	0.65 (–0.25 to 1.54) Moderate
			*0.025*	0.34 (–0.54 to 1.23) Small
			*0.045*	0.28 (–0.60 to 1.16) Small
Push-ups (reps)	*Time effect*			
PT + BA	48.8 ± 4.6	53.1 ± 5.3	*0.001*	0.83 (–0.08 to 1.74) Moderate
PT + PL	49.0 ± 4.1	52.2 ± 3.9	*0.001*	0.77 (–0.14 to 1.67) Moderate
CON	48.8 ± 3.6	49.5 ± 3.5	*0.774*	0.19 (–0.69 to 1.07) Trivial
*Post-test comparisons*	*Group x time effect, p = 0.001, n2p = 0.85*			
	PT + BA vs. CON PT + PL vs. CON PT + BA vs. PT + PL		*0.006*	0.77 (–0.14 to 1.68) Moderate
			*0.027*	0.70 (–0.20 to 1.60) Moderate
			*0.041*	0.19 (–0.69 to 1.06) Trivial
1RM bench press (kg)	*Time effect*			
PT + BA	68.0 ± 7.9	73.5 ± 8.7	*0.001*	0.63 (–0.26 to 1.53) Moderate
PT + PL	68.5 ± 7.8	72.3 ± 8.4	*0.001*	0.45 (–0.44 to 1.34) Small
CON	68.3 ± 9.2	69.0 ± 9.1	*0.657*	0.07 (–0.80 to 0.95) Trivial
*Post-test comparisons*	*Group x time effect, p = 0.001, n2p = 0.88*			
	PT + BA vs. CON PT + PL vs. CON PT + BA vs. PT + PL		*0.011*	0.48 (–0.41 to 1.37) Small
			*0.027*	0.36 (–0.61 to 1.37) Small
			*1.000*	0.13 (–0.74 to 1.01) Trivial

**Table 4.** Changes in the power performance from pre to post-training (mean ± SD).

Variable	Pre	Post	Significance	ES (95% CI)
PPO (w)			*Time effect*	
PT + BA	227.0 ± 10.8	244.3 ± 10.0	*0.001*	1.59 (0.59 to 2.60) Large
PT + PL	225.6 ± 12.2	234.9 ± 12.9	*0.001*	0.71 (–0.19 to 1.61) Moderate
CON	226.6 ± 11.4	227.8 ± 10.7	*0.911*	0.10 (–0.77 to 0.98) Trivial
*Post-test comparisons*			*Group x time effect, p = 0.001, n2p = 0.90*	
	PT + BA vs. CON PT + PL vs. CON PT + BA vs. PT + PL		*0.001*	1.54 (0.55 to 2.54) Large
			*0.006*	0.60 (–0.32 to 1.47) Moderate
			*0.026*	0.81 (–0.13 to 1.69) Moderate
MPO (w)			*Time effect*	
PT + BA	146.0 ± 9.8	155.6 ± 9.6	*0.001*	0.95 (0.02 to 1.87) Moderate
PT + PL	145.6 ± 12.2	151.5 ± 12.3	*0.011*	0.46 (–0.43 to 1.35) Small
CON	146.6 ± 11.4	147.7 ± 11.5	*0.841*	0.09 (–0.78 to 0.97) Trivial
*Post-test comparisons*			*Group x time effect, p = 0.001, n2p = 0.86*	
	PT + BA vs. CON PT + PL vs. CON PT + BA vs. PT + PL		*0.001*	0.75 (–0.19 to 1.62) Moderate
			*0.019*	0.32 (–0.58 to 1.19) Small
			*0.033*	0.37 (–0.53 to 1.24) Small

**Table 5.** Changes in the swimming performance variables from pre to post-training (mean ± SD).

Variable	Pre	Post	Significance	ES (95% CI)
50-m freestyle (sec)	*Time effect*			
PT + BA	25.8 ± 1.1	25.2 ± 1.1	*0.026*	–0.52 (–1.41 to 0.37) Small
PT + PL	25.7 ± 1.3	25.4 ± 1.3	*0.046*	–0.23 (–1.10 to 0.66) Small
CON	25.6 ± 1.2	25.5 ± 1.2	*0.991*	–0.08 (–0.96 to 0.80) Trivial
*Post-test comparisons*	*Group x time effect, p = 0.001, n2p = 0.81*			
	PT + BA vs. CON PT + PL vs. CON PT + BA vs. PT + PL		*0.023*	–0.26 (–1.13 to 0.63) Small
			*0.054*	–0.08 (–0.95 to 0.80) Trivial
			*0.574*	–0.16 (–1.04 to 0.72) Trivial
100-m freestyle (sec)	*Time effect*			
PT + BA	56.8 ± 1.4	56.0 ± 1.5	*0.011*	–0.53 (–1.42 to 0.36) Small
PT + PL	56.8 ± 1.6	56.3 ± 1.6	*0.038*	–0.30 (–1.18 to 0.58) Small
CON	56.5 ± 1.4	56.4 ± 1.4	*0.852*	–0.07 (–0.95 to 0.81) Trivial
*Post-test comparisons*	*Group x time effect, p = 0.001, n2p = 0.78*			
	PT + BA vs. CON PT + PL vs. CON PT + BA vs. PT + PL		*0.033*	–0.27 (–1.13 to 0.63) Small
			*0.655*	–0.07 (–0.94 to 0.81) Trivial
			*0.423*	–0.19 (–1.06 to 0.69) Trivial
200-m freestyle (sec)			*Time effect*	
PT + BA	124.7 ± 3.1	123.6 ± 3.3	*0.039*	–0.33 (–1.21 to 0.55) Small
PT + PL	124.5 ± 2.7	123.4 ± 2.6	*0.044*	–0.40 (–1.28 to 0.49) Small
CON	124.4 ± 2.3	124.2 ± 2.4	*0.985*	–0.08 (–0.96 to 0.80) Trivial
*Post-test comparisons*	*Group x time effect, p = 0.001, n2p = 0.81*			
	PT + BA vs. CON PT + PL vs. CON PT + BA vs. PT + PL		*0.017*	–0.21 (–1.08 to 0.68) Small
			*0.022*	–0.32 (–1.19 to 0.58) Small
			*1.000*	*–0.07 (–0.94 to 0.81) Trivial*

**Table 6.** Changes in the stroke rate performance from pre to post-training (mean ± SD).

Variable	Pre	Post	Significance	ES (95% CI)
Stroke rate 50-m (cycle.min)^−1^	*Time effect*			
PT + BA	54.8 ± 2.0	53.3 ± 1.9	*0.044*	–0.74 (–1.64 to 0.17) Moderate
PT + PL	54.7 ± 2.1	54.1 ± 2.2	*0.127*	–0.27 (–1.15 to 0.61) Small
CON	54.5 ± 1.6	54.2 ± 1.5	*0.741*	–0.19 (–1.06 to 0.69) Trivial
*Post-test comparisons*	*Group x time effect, p = 0.001, n2p = 0.52*			
	PT + BA vs. CON PT + PL vs. CON PT + BA vs. PT + PL		*0.009*	–0.53 (–1.39 to 0.39) Small
			*0.111*	–0.05 (–0.93 to 0.83) Trivial
			*0.989*	–0.39 (–1.04 to 0.72) Small
Stroke rate 100-m (cycle.min)^−1^			*Time effect*	
PT + BA	51.8 ± 2.1	50.6 ± 1.5	*0.037*	–0.63 (–1.53 to 0.27) Moderate
PT + PL	51.7 ± 2.8	51.0 ± 2.5	*0.227*	–0.25 (–1.13 to 0.63) Small
CON	51.8 ± 2.2	51.4 ± 1.7	*0.851*	–0.19 (–1.07 to 0.67) Trivial
*Post-test comparisons*	*Group x time effect, p = 0.003, n2p = 0.35*			
	PT + BA vs. CON PT + PL vs. CON PT + BA vs. PT + PL		*0.026*	–0.50 (–1.37 to 0.41) Small
			*0.678*	–0.19 (–1.06 to 0.70) Trivial
			*0.914*	–0.19 (–1.06 to 0.69) Trivial
Stroke rate 200-m (cycle.min)^−1^			*Time effect*	
PT + BA	44.9 ± 2.7	44.0 ± 2.6	*0.041*	–0.33 (–1.21 to 0.56) Small
PT + PL	44.7 ± 1.6	44.2 ± 1.5	*0.322*	–0.31 (–1.19 to 0.57) Small
CON	44.5 ± 2.1	44.4 ± 1.9	*1.000*	–0.05 (–0.92 to 0.83) Trivial
*Post-test comparisons*	*Group x time effect, p = 0.001, n2p = 0.42*			
	PT + BA vs. CON PT + PL vs. CON PT + BA vs. PT + PL		*0.039*	–0.18 (–1.05 to 0.71) Trivial
			*0.860*	–0.12 (–0.99 to 0.77) Trivial
			*0.901*	–0.09 (–0.97 to 0.79) Trivial

**Table 7.** Changes in the immunoendocrine variables from pre to post-training (mean ± SD).

Variable	Pre	Post	Significance	ES (95% CI)
Testosterone (μg·dL)^−1^	*Time effect*			
PT + BA	0.60 ± 0.10	0.62 ± 0.10	*0.041*	0.21 (–0.68 to 1.08) Small
PT + PL	0.60 ± 0.08	0.60 ± 0.09	*1.000*	0.00 (–0.88 to 0.88) Trivial
CON	0.62 ± 0.08	0.61 ± 0.08	*1.000*	–0.12 (–1.00 to 0.76) Trivial
*Post-test comparisons*	*Group x time effect, p = 0.001, n2p = 0.60*			
	PT + BA vs. CON PT + PL vs. CON PT + BA vs. PT + PL		*0.033*	0.11 (–0.77 to 0.98) Trivial
			*0.955*	–0.12 (–0.99 to 0.76) Trivial
			*0.011*	0.21 (–1.68 to 1.08) Small
Cortisol (μg·dL)^−1^	*Time effect*			
PT + BA	19.8 ± 1.8	19.0 ± 1.7	*0.022*	–0.44 (–1.32 to 0.45) Small
PT + PL	19.5 ± 4.1	19.8 ± 4.1	*0.844*	0.07 (–0.81 to 0.95) Trivial
CON	19.6 ± 4.1	19.6 ± 4.1	*1.000*	0.00 (–0.88 to 0.88) Trivial
*Post-test comparisons*	*Group x time effect, p = 0.001, n2p = 0.69*			
	PT + BA vs. CON PT + PL vs. CON PT + BA vs. PT + PL		*0.033*	–0.19 (–1.06 to 0.70) Trivial
			*0.678*	0.05 (–0.83 to 0.92) Trivial
			*0.028*	–0.25 (–1.12 to 0.64) Small
IgA (mg/L)		*Time effect*		
PT + BA	174.6 ± 25.1	174.1 ± 24.1	*1.000*	–0.02 (–0.90 to 0.86) Trivial
PT + PL	177.3 ± 26.7	171.5 ± 26.5	*0.039*	–0.22 (–1.09 to 0.67) Small
CON	172.2 ± 29.8	171.3 ± 29.5	*0.911*	–0.03 (–0.91 to 0.85) Trivial
*Post-test comparisons*	*Group x time effect, p = 0.001, n2p = 0.55*			
	PT + BA vs. CON PT + PL vs. CON PT + BA vs. PT + PL		*0.841*	0.10 (–0.78 to 0.98) Trivial
			*0.860*	–0.01 (–0.88 to 0.87) Trivial
			*0.042*	0.10 (–0.78 to 0.98) Trivial

### 3.3. BA vs. PL effects

**Figure 5.**
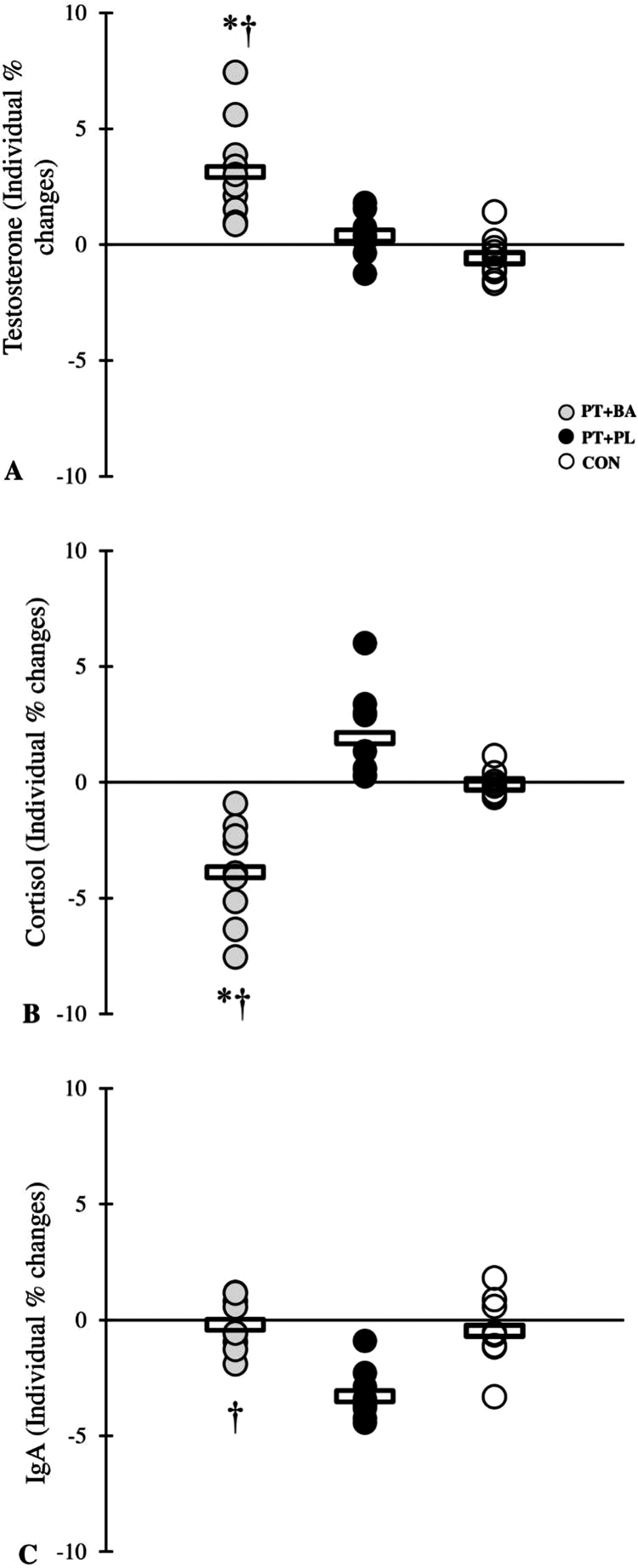
% changes in the immunoendocrine through the 8-week intervention period. *denotes significant differences compared with the CON, †denotes significant differences compared with the PL.

Post hoc analyses revealed that the BA group demonstrated greater improvements than the PL group in MBT (*p* = 0.045), push-ups (*p* = 0.041), PPO (*p* = 0.026), MPO (*p* = 0.033), and also more changes in the testosterone (*p* = 0.011), cortisol (*p* = 0.028) and IgA (*p* = 0.042) (Figure 5).

## 4. Discussion

The present study is the first to examine the combined effects of an upper-body PT program and BA supplementation on physical performance, swimming performance, and immunoendocrine responses in trained male swimmers. The primary findings were that: (i) both PT groups elicited significant improvements in power, strength, anaerobic power, muscular endurance, and freestyle swimming performance compared with the CON group; (ii) BA supplementation augmented several PT-induced adaptations, particularly for MBT performance, push-ups, PPO and MPO, and swimming performance; and (iii) BA supplementation elicited a more favorable immunoendocrine profile, characterized by increased testosterone, reduced cortisol, and maintenance of IgA concentrations. Although the present findings suggest that BA supplementation may enhance the adaptive responses to upper-body PT, these results should be interpreted cautiously due to the relatively short intervention period and the multifactorial nature of training adaptations in swimmers. Collectively, these findings indicate that BA supplementation may support the effectiveness of upper-body PT by contributing to performance adaptations and physiological homeostasis during periods of intensified training.

### 4.1. Performance adaptations to PT

Consistent with the study hypothesis and previous evidence in non-swimming populations, the implementation of upper-body PT induced meaningful improvements in muscular power (MBT), maximal strength (1RM), muscular endurance (push-ups), and anaerobic power (PPO and MPO) [4,5,7–9,23]. These adaptations likely reflect enhanced neuromuscular function, including improved motor unit recruitment, firing frequency, and intermuscular coordination, which are hallmark adaptations to plyometric-type loading [8,23]. Although PT has been extensively studied in different sport populations [7,8], its application in swimming has been largely neglected [5]. Therefore, the present findings provide novel empirical support for the efficacy of upper-body PT as a complementary training strategy for swimmers.

Importantly, the observed improvements in 50-, 100-, and 200-m freestyle performance suggest meaningful transfer of dry-land neuromuscular adaptations to in-water performance. Upper-body power and strength are critical determinants of propulsion during sprint- and middle-distance freestyle swimming, particularly during the pull phase and at higher stroke rates [1–3]. Enhanced rate of force development and power output may allow swimmers to generate greater propulsive force per stroke, thereby improving swimming velocity and efficiency [3]. However, these improvements should not be interpreted as being exclusively attributable to PT, as swimming performance is influenced by multiple physiological and technical factors, including swimming technique, aerobic conditioning, and overall training load. These findings extend previous correlational evidence linking upper-body power to swimming performance by demonstrating that a structured PT intervention may positively contribute to swimming-specific performance adaptations [2].

### 4.2. Augmenting effects of BA on performance adaptations

A key finding of the present study was that BA supplementation augmented several PT-induced adaptations compared with PL, particularly for anaerobic power (PPO and MPO), upper-body muscular power and endurance, and swimming performance. These findings align with the established ergogenic role of BA in enhancing intramuscular carnosine concentrations, thereby improving intracellular buffering capacity during high-intensity exercise [9,10]. *Because upper-body PT imposes repeated high-intensity muscular contractions with substantial glycolytic demand, the accumulation of hydrogen ions during training may contribute to fatigue and reductions in movement quality [4,5].* In this context, enhanced buffering capacity associated with BA supplementation may have enabled participants to better tolerate the metabolic stress of repeated explosive upper-body actions, thereby helping to maintain training quality throughout the intervention period [10].

The greater improvements in PPO and MPO observed in the BA group are particularly noteworthy, as these variables reflect the capacity to sustain high power output during metabolically demanding exercise [15]. While neuromuscular adaptations alone may explain improvements in explosive tasks such as MBT and power output, the superior adaptations observed following BA supplementation likely reflect the combined influence of neuromuscular and metabolic factors [24]. *Nevertheless, given the short duration of the intervention and the absence of direct measurements of muscle carnosine concentration or intracellular buffering activity, the proposed physiological mechanisms remain speculative and should be interpreted with caution.*

*It is also plausible that improved buffering capacity reduced the perception of fatigue and allowed swimmers to maintain greater movement velocity and technical execution during repeated PT sessions, which may have contributed to the superior adaptations observed in the BA group [9,10].* These findings support the notion that BA supplementation may be particularly beneficial when training programs involve repeated high-intensity upper-body efforts, as is characteristic of PT.

### 4.3. Immunoendocrine responses to training

In addition to performance outcomes, the current study offers new insights into the immunoendocrine responses resulting from the combined application of PT and BA supplementation in swimmers. The BA group showed an increase in testosterone levels and a decrease in cortisol levels after the intervention, whereas the PL group demonstrated a decline in testosterone and an increase in cortisol. Furthermore, IgA levels remained stable in the BA group but were significantly reduced in the PL group. Collectively, these findings suggest that BA supplementation may contribute to a more favorable anabolic–catabolic hormonal balance and reduced immune perturbations associated with training stress.

Increases in cortisol and decreases in IgA are frequently associated with excessive training stress, impaired recovery, and a heightened risk of illness among swimmers [13]. The preservation of IgA levels in the BA group may indicate that improved buffering capacity attenuated some of the physiological stress associated with repeated high-intensity training demands [15]. *However, because immunoendocrine responses are influenced by several factors, including nutrition, sleep, and overall training load, these findings should be interpreted carefully.* While the exact mechanisms linking BA supplementation to immunoendocrine regulation remain unclear, diminished metabolic acidosis and reduced physiological stress responses may partially explain these effects [24]. Significantly, these results highlight the potential role of BA not only as a performance-enhancing supplement but also as a strategy that may improve recovery capacity and tolerance to intensive training loads during the preparation phase in swimmers.

### 4.4. Limitations and future directions

*Several limitations of the present study should be acknowledged when interpreting the findings. First, the duration of the intervention was relatively short, which may limit the ability to fully capture long-term physiological and performance adaptations associated with upper-body PT and BA supplementation. Second, muscle carnosine concentration was not directly measured; therefore, the proposed buffering-related mechanisms underlying the ergogenic effects of BA remain inferential. Third, the sample consisted exclusively of trained male swimmers, which may restrict the generalizability of the results to female athletes, younger swimmers, or athletes from other competitive levels.*

*Despite these limitations, the study provides novel insights into the combined effects of upper-body PT and BA supplementation in swimmers. Future research should include longer intervention periods and incorporate direct assessments of muscle carnosine content to better clarify the mechanistic role of BA supplementation. In addition, future studies should examine similar interventions in female swimmers and athletes from different performance levels to determine whether comparable adaptations occur across populations. Further investigations may also explore how improved buffering capacity influences training quality, fatigue resistance, and recovery responses during repeated high-intensity swimming and dry-land training sessions.*

## 5. Conclusions

In summary, an 8-week PT regimen focused on the upper body markedly improved physical performance, anaerobic power, and freestyle swimming capabilities in trained male swimmers. Notably, the supplementation of β-alanine further enhanced several of these adaptations and resulted in a more advantageous immunoendocrine profile, which was marked by elevated testosterone levels, decreased cortisol, and stable IgA concentrations. These results offer new evidence that supports the synergistic application of upper-body plyometric training and β-alanine supplementation as an effective approach to boost performance and physiological resilience in competitive swimmers.
